# Effects of Exergames on Motor Skills, Psychological Well-Being, and Cognitive Abilities in Schoolchildren and Adolescents: Scoping Review

**DOI:** 10.2196/71416

**Published:** 2025-09-17

**Authors:** Eleonora Rosi, Valentina Bianchi, Ilaria Baù, Rebecca Nuzzo, Stefania Valsecchi, Massimo Molteni, Paola Colombo

**Affiliations:** 1 Child Psychopathology Unit-Smart Lab, IRCCS Eugenio Medea, Via Don Luigi Monza, 20, Bosisio Parini, 23842, Italy, 39 031877619

**Keywords:** exergames, active video games, physical education, physical activity, school, children, adolescents

## Abstract

**Background:**

In a world where children are increasingly sedentary, the need for innovative solutions to promote physical activity is felt more than ever. Exergames—interactive video games combining physical activity with gaming—are an attractive way to engage children in exercise while having fun. Although exergames have demonstrated several benefits for the health and physical activity of children and young people, the impact of these devices is poorly explored, especially in the school context.

**Objective:**

This scoping review was aimed at analyzing the effects of exergames on motor skills, psychological well-being, and cognitive abilities in children and adolescents during physical education hours and play-based activities. Our specific goal was to explore and describe the effects of exergames in school programs and their potential to improve physical and mental health in educational settings.

**Methods:**

We carried out our review in accordance with the PRISMA-ScR (Preferred Reporting Items for Systematic Reviews and Meta-Analyses Extension for Scoping Reviews) guidelines. We searched 3 bibliographic databases from 2019 to June 2024 and included all scientific studies involving children and adolescents interacting with exergames during physical education lessons at school.

**Results:**

Our database search produced 1694 articles. After performing 3 levels of screening (title, abstract, and full text), 25 articles were left. The majority of the studies confirmed that the use of exergames during physical education and playful sport activities is associated with a number of improvements. More specifically, these devices can provide motor benefits as well as psychological or cognitive benefits, such as cognitive flexibility and attentional functions, overall well-being, and a greater sense of self-efficacy, self-confidence, and mood.

**Conclusions:**

These results may have significant implications for public health or education: exergames may become accessible and useful devices for promoting physical activity in young people, potentially benefiting motor skills but also psychological and cognitive functions, increasing children’s participation in physical activities, and leading to a general improvement in their sense of self-efficacy and well-being. Exergames can improve children’s physical and cognitive skills, thus becoming a complementary and additional device to traditional physical education exercises and helpful tools to increase physical movement in extracurricular activities.

## Introduction

The World Health Organization (WHO) has underlined the importance of physical activity, which is beneficial to health and mental and physical well-being, for the prevention of noncommunicable diseases [1]. Insufficient levels of physical exercise or physical inactivity may contribute to the development of diseases, such as obesity and overweight, cardiovascular disease, and motor development–related disorders [2,3]. Especially in children and adolescents, sedentary behavior increased adiposity and led to poorer cardiometabolic health, fitness, and behavioral conduct or prosocial behavior, as well as reduced sleep duration [1]. According to data from the Italian Institute of Statistics for the 2017‐2018 period, Italian children and adolescents did not engage in sufficient levels of physical activity [4] as recommended by the WHO, whereby they should practice at least 60 minutes of moderate to vigorous physical activity for 3 days in a week [5,6]. In addition, Italian Institute of Statistics data (2021) highlighted that continuous sporting activity in children and young people aged 3‐17 years has decreased significantly, from 51.3% to 36.2%, while sedentariness has increased (from 22.3% to 27.2%) [7]. This trend is in line with other countries [8-10].

In accordance with the above-mentioned data, schools are considered the main educational context for the promotion of physical activity. There are multiple opportunities for exercise in the context of school, such as during recess, sports, physical education (PE) lessons, and active movement to and from school [11]. Several studies have shown that school-based focused interventions have been effective in increasing children’s physical activity levels, achieving up to 50% of the weekly requirement identified by the WHO [1,12].

PE lessons aim to promote motivation and physical exercise in students [12]. However, the role of PE in the school system has led to some criticism concerning the methods and tools that are currently being used during classes, which generate very low interest among pupils and students [13]. With specific reference to educational processes connected to physical and sporting activity, the scientific community is now investigating the effects of incorporating devices into traditional teaching in order to increase student motivation and participation [14].

Among the technological tools available today, there are exergames. Their introduction in everyday life contexts not only helps children and young people to achieve the recommended levels of physical activity but also has a positive effect on their lives, as exergames foster the acquisition and development of motor skills and abilities, increasing participation in sporting activity [15].

Exergames—also known as active video games—are an activity combining video games and motor exercise and requiring physical effort on the part of the child [16]. They are characterized by the use of audio or visual feedback on performance compared to traditional physical activity, thus making it more enjoyable [16]. These digital motor activities aim to stimulate motor and motivational skills [12].

Exergames are based on the conversion of real movements within a virtual environment, allowing players to be more active by practicing virtual sports, fitness exercises, and other playful physical activities [15].

In addition to making health-promoting behavior more attractive and desirable, exergames also offer numerous advantages, such as increased motivation and engagement and a greater possibility for fun and challenge [17]. Their pros are also evident when they are compared with traditional exercises [18]. Exergames are more engaging, increasing motivation to train and consequently improving both motor skills, such as balance and mobility [17], and cognitive skills [18,19]. Moreover, they allow players to focus more on the impact of their own movements during the game and less on movements for their own sake [20]. Finally, they are easily adaptable to various contexts—they require a console and screen or projector—and can be carried out either alone or in small groups, encouraging collaboration and cooperation [18].

As claimed by previous reviews, exergames are able to promote a physically active lifestyle in children and young people [21,22]. However, most of the previous reviews have focused on exergame use in specific contexts and situations, such as nutrition and obesity [23-25], chronic diseases [26,27], and physical activity in general [15,28-30]. Specifically, Lamas and colleagues [21] have evaluated the effectiveness of using exergames to promote nutrition education and physical activity in children and adolescents. Their review points out the effectiveness of using exergames to promote health and prevent obesity by interventions aimed at increasing children’s and adolescents’ nutritional knowledge and introducing healthier and more balanced diets [21]. Other meta-analyses have focused on the benefits of exergames in school settings, specifically regarding PE learning. In particular, they have shown that exergames can improve performance in PE education and suggest using exergames in small classes, limiting the implementation cycle to 1‐2 months, and selecting games according to different age groups [31].

Although exergames have demonstrated various benefits concerning the health and physical activity of children and young people, no review has been carried out so far on their potential use and benefits in the school setting, exploring not only motor or physical effects but also psychological and cognitive effects.

Therefore, the aim of this study was to expand and update knowledge on the potential effects of exergames within the school context, considering the past 5 years. A scoping review was conducted to illustrate and analyze the effectiveness of the use of exergames in schools (children aged 6‐18 years) during PE lessons and play or sports activities, to understand their benefits and advantages, and their future application as inclusive tools.

## Methods

This review was conducted according to the PRISMA-ScR (Preferred Reporting Items for Systematic Reviews and Meta-Analyses Extension for Scoping Reviews) guidelines [32]. Four authors (IB, RN, ER, and SV) searched 3 bibliographic databases from 2019 to June 30, 2024: PubMed, Web of Science, and Scopus. The definitive search strategy is the following string: (“physical exercise” OR “physical education”) AND (“exergaming” OR “active video games” OR “exergames” OR “interactive videogame” OR “technology”) AND (“children” OR “adolescent”) AND (“school” OR “school education” OR “education”) NOT (meta-analysis OR review). For complete details of the search strings, refer to Multimedia Appendix 1. The search was manually completed, following the PCC (population, concept, and context) format (Table 1). We included all scientific studies involving children and adolescents, aged between 6 and 18 years, interacting with exergames during PE hours. We excluded papers not written in English, reviews and meta-analyses, and articles on adult participants or animals. We also excluded studies that used other types of technologies during PE hours or technologies with an evaluative purpose of sports performance and studies not related to the school setting.

**Table 1.** PCC (population, concept, and context) format.

Components	Description
Population (P)	Children and adolescents aged 6-18 years
Concept (C)	Experimental study with random or nonrandomized assignment, quasi-experimental study, cross-sectional study, natural experiment, and uncontrolled trial within exergames to assess motor, psychological, and cognitive skills Exergames—devices using a digital game-based exercise system (eg, consoles such as Xbox 360 Kinect, HOPSports Brain Breaks, and Nintendo Wii Fit)
Context (C)	Exergames during physical education lessons and other physical activity hours at school

All record titles and abstracts retrieved from the database search were screened by 4 blinded reviewers (IB, RN, ER, and SV), who excluded studies that did not meet the eligibility criteria. The same authors proceeded with the full-text screening of retained papers according to the inclusion criteria. In case of discordant opinions, the 4 authors voted to reach a decision. Interrater agreement was evaluated with the Fleiss kappa statistic (κ) [33,34]. Data from the articles were extracted into an extraction table, which includes columns for authors and year of publication; sample size, age, experimental groups, and country; methodology and duration of the sessions; outcome measures; and results.

## Results

**Figure 1.**
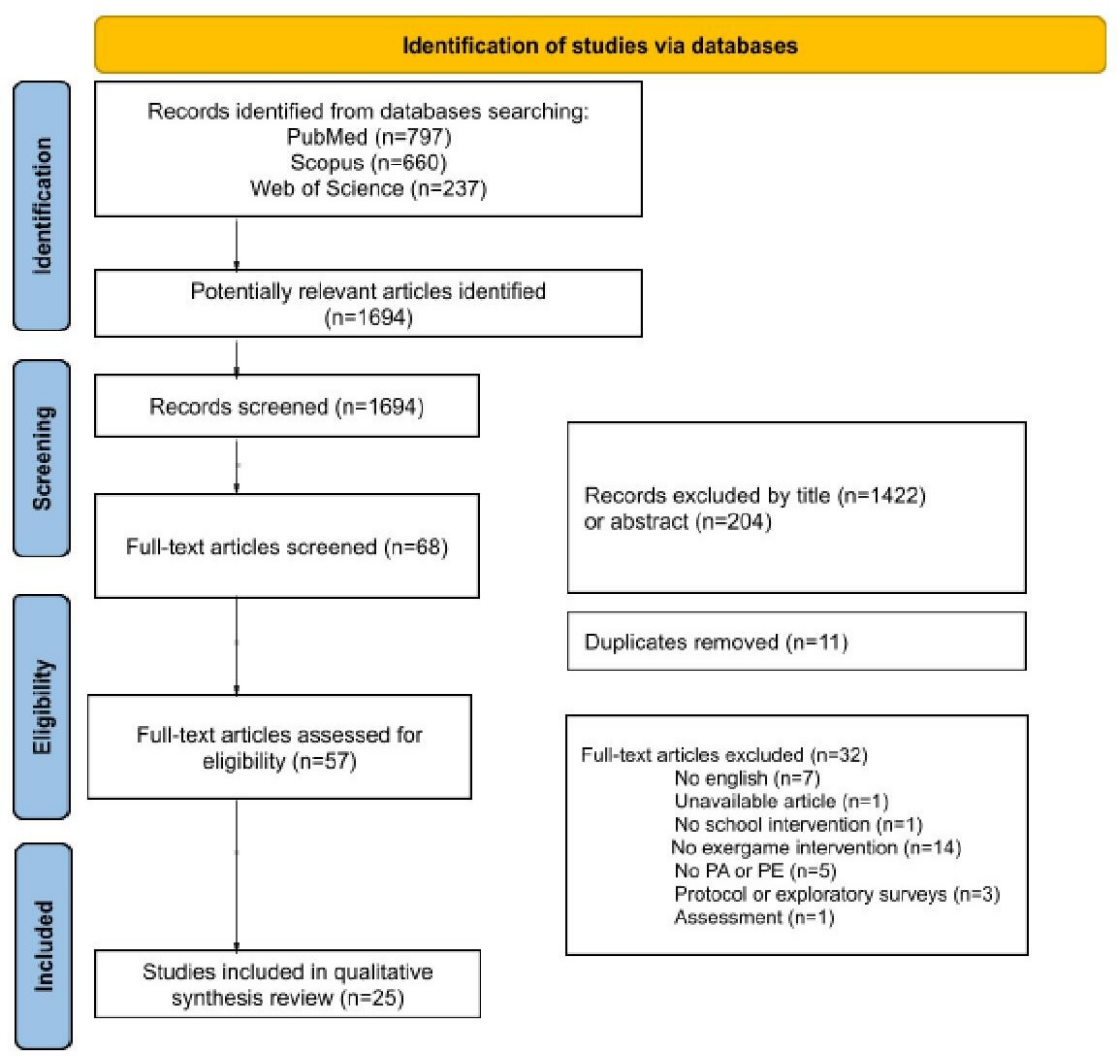
PRISMA-ScR (Preferred Reporting Items for Systematic Reviews and Meta-Analyses Extension for Scoping Reviews) flow diagram. PA: physical activity; PE: physical education.

Our search from 3 databases produced 1694 articles. After screening titles and abstracts and removing duplicates, there were 57 articles. A total of 25 articles were included in this review. The PRISMA-ScR flowchart is shown in Figure 1. The interrater reliability for full-text screening was substantial across authors, with κ=0.65, κ=0.72, κ=0.68, and κ=0.71 [33,34].

Table 2 summarizes the 25 studies included in this review, that is, research that used exergames within PE school hours. In two cases, the sample of study is the same but with different statistical analyses and research objectives. They are indicated by footnotes in the table.

**Table 2.** Studies comprising the use of exergames in physical education during school hours.

Reference	Sample, age, country, and groups	Methodology and durations	Outcome measures	Results
Andrade et al (2019)^a^ [35]	140 participants (59 males) Age range: 7‐11 years (mean 9.41, SD 0.68 years) Country: Brazil EG^b^: 68 students (mean age 8.85, SD 0.44 years) CG^c^: 72 students (mean age 9.96, SD 0.31 years)	Nonrandomized controlled study 1 week EG intervention: three 40-minute sessions with Just Dance 2015 exergame, Xbox 360 Kinect console CG intervention: 3 regular PE^d^ classes	Anthropometric measures: weight (kg) and height (m), BMI (kg/m^2^) BRUMS^e^ to assess mood in 6 dimensions: 3 psychological states (feeling of depression, anger, and mental confusion) and 3 psychosomatic states (fatigue, tension, and vigor)	Posttest, EG tension and mental confusion reduced, vigor increased Significant time effect on mental confusion and tension for all participants Significant group difference on vigor (higher EG than CG) Significant time-group effect on fatigue
Andrade et al (2020)^a^ [36]	140 participants (59 males) Age range: 7‐11 years (mean 9.41, SD 0.48 years) Country: Brazil EG: 68 students (male: mean age 8.77, SD 0.58 years; female: mean age 8.88, SD 0.39 years) CG: 72 students (male: mean age 9.46, SD 0.77 years; female: mean age 9.38, SD 0.62 years)	Cluster-randomized natural experiment 1 week EG intervention: three 40-minute sessions with Just Dance 2015 exergame, Xbox 360 Kinect console CG intervention: 3 regular PE classes	Anthropometric measures: weight (kg) and height (m), BMI (kg/m^2^) BRUMS to assess mood in 6 dimensions: 3 psychological states (feeling of depression, anger, and mental confusion) and 3 psychosomatic states (fatigue, tension, and vigor) Rosenberg Self-Esteem Scale	Posttest, higher tension in EG boys than in CG boys; vigor is higher among girls from the EG compared to the CG EG boys had higher levels of anger than girls CG boys had lower scores for mental confusion than girls CG boys have lower anger, mental confusion, and higher self-esteem than EG boys EG girls have moderate magnitudes, higher vigor, and lower mental confusion vs CG
Balasekaran et al (2021) [37]	113 participants (47 males) Age range: 8‐11 years (mean 9.68, SD 0.95 years) Country: Singapore EG: 48 students (mean age 9.71, SD 0.99 years) CG: 65 students (mean age 9.66, SD 0.94 years)	Quasi-experimental study 10 weeks EG intervention: 3‐5 minutes daily during class time 5 days per week with HOPSports Brain Breaks, Physical Activity Solutions’ videos Use of video projectors CG intervention: usual academic activity	APAS^f^ to assess beliefs, attitudes, and self-efficacy PA^g^ (test pre-post)	Significant increase in the mean APAS scores for both groups Significant group effect on importance, learning, self-efficacy, fun, and fitness Significant time-group interaction effects for all APAS scores Increase APAS scores in EG at posttest vs CG Significant gender difference (males have higher self-efficacy) in post-F3 for EG
Bonnema et al (2022) [38]	122 participants (42 males) Age range: 11‐12 years (mean 11.92, SD 0.36 years) Country: South Africa EG: 73 students (mean age 11.85, SD 0.38 years) CG: 49 students (mean age 12.02, SD 0.31 years)	Nonrandomized intervention study Daily PE intervention for 12 weeks EG intervention: HOPSports Brain Breaks + standard PE CG intervention: no video program, standard PE	Eurofit test to assess physical fitness 20 m SRT^h^ for cardiovascular endurance 10 × 5 m shuttle test for running speed, agility Plate tapping test for speed of limb movement Stork balance test for balance Sit-and-reach test for hamstring flexibility Standing long jump test for abdominal strength Anthropometric measures: body mass, stature, skinfolds, and BMI	EG improve in all the physical fitness components Significant differences between the EG and CG groups in body fat, stork balance, plate tapping, sit-and-reach, standing jump, sit-ups, 10 × 5 m shuttle run, and 20 m shuttle run
Chae et al (2022) [39]	109 participants (50 males) Age range: 15‐17 years (male: mean 16, SD 0.71 years; female: mean 15.7, SD 0.61 years) Country: Republic of Korea EG: 50 students CG: 59 students	Nonequivalent control group with a nonsynchronized pretest-posttest design 12 weeks EG intervention: 30 minutes, 5 times a week (3 during lunch break and 2 during PE class) Exergame: Wii Fit Console: Nintendo Wii Sports (bowling, boxing, tennis, cycling, table tennis, and basketball) CG intervention: usual activity, no intervention	Sociodemographic questionnaire self-reported Anthropometric measures: BMI (InBody J10); waist circumference (Tanita retractable ruler) Physiological factor: Cholestech LDX Analyzer Blood test: lipids, HDL^i^, LDL^j^, total cholesterol, triglyceride, and glucose level Pedometer: Fitbit Zip (daily steps) in school Weekday sitting time Program satisfaction using 5 open-ended questions	Significant differences between the groups in skeletal muscle mass, waist circumference, HDL-cholesterol, LDL-cholesterol, and weekend sitting time Significant group and time interactions were found for skeletal muscle mass and waist circumference HDL-cholesterol increased EG decreased in CG, with a significant between-group difference LDL levels increased in CG, with a significant between-group difference Weekend daily sitting time significantly decreased in EG compared with CG
Dantas et al (2022) [40]	32 participants (males not specified) Age range: 8‐10 years Country: Brazil EG: 22 students (mean age 8.73, SD 0.76 years) CG: 10 students (mean age 8.30, SD 0.67 years)	Quasi-experimental research Randomized groups Evaluation before and after 16 weeks Duration of the intervention is not specified EG intervention: Just Dance 2015 exergame (20 minutes); Microsoft Xbox One console; Kinect accessory CG intervention: routine activities planned	Sit-and-reach test (Wells and Dillon) to assess flexibility Arm flexion and abdominal tests to assess strength and resistance Body weight	Significant changes in flexibility for EG EG presented significant adaptation for the upper limb strength There were no significant changes in the variable abdominal strength
García-Massó et al (2023) [41]	30 participants (16 male) Age range: 12‐14 years Country: Spain EHCLG^k^: 10 students (mean age 13, SD 1 year) ELCLG^l^: 10 students (mean age 13, SD 1 year) CG: 10 students (mean age 13, SD 1 year)	Quasi-experimental study 2 weeks EHCLG intervention: two 45-minute sessions per week Exergame: Feline Runner, Hungry Monster, Whack-a-Slime Wii Balance Board, LabVIEW, and 15ʺ laptop ELCLG intervention: two 45-minute sessions per week Exergame: Feline Runner, Hungry Monster, Whack-a-Slime Wii Balance Board, LabVIEW, and 15ʺ laptop CG intervention: traditional PE	Five tests (quiet standing with eyes open and eyes closed, forward limit of stability, pattern tracking in an anterior-posterior direction, and pattern tracking in medial-lateral direction) to assess postural control and stability on the Wii Balance Board	Significant differences between groups after the intervention “Forward limit of stability” was significantly higher in the group who trained with high-cognitive-load exergames compared with the group who trained with traditional physical education exercises Significant intragroup differences between the initial and final assessments for the training groups assigned to both high- and low-cognitive-load exergames Significant difference between pre- and postassessments after training in low-cognitive-load exergames, only in the anterior-posterior axis
Goncalves et al (2024) [42]	79 children (34 girls and 45 boys) Age range: 7‐11 years (mean 8.91, SD 1.21 years) Country: France EG: 79 children	Pilot interventional study Pre-post design 3 weeks Habituation phase: 4 sessions of 30 minutes, 1 week apart Intervention: 30-minute DPA^m^ sessions using Play LÜ Exergame platform combined with a motor skills circuit	PLAYself tool to assess physical literacy Academic performance measured using standardized math and French tests from the French Ministry of Education Soft skills (motivation, self-efficacy, and concentration) evaluated through adapted, brief questions on a visual analog scale completed by students, and through short interviews with teachers who rated observed changes in their classrooms on a 1 to 10 scale	Increased scores for physical literacy Increased motivation in mathematics General improvement in concentration in class Higher academic achievement, concentration, and self-efficacy in French No significant changes observed in academic performance or self-efficacy in mathematics
Irandoust et al (2021) [43]	59 participants with obesity (59 males) Age range: not specified Country: Iran VGG^n^: 21 students (mean age 8.91, SD 1.21 years) AEG^o^: 18 students (mean age 9.30, SD 1.30 years) CG: 20 students (mean age 8.95, SD 1.15 years)	Randomized, controlled, single-blinded study 12 weeks VGG intervention: 3 sessions of 60 minutes per week Exergame: Wii Sports, Kinect Ultimate Sports, Wii Fit, and Just Dance using the Xbox Kinect game AEG intervention: 3 sessions of 60‐70 minutes per week CG intervention: usual PA	Anthropometrics measures: height (cm), weight (kg), body fat (%), WHR^p^, and BMI (kg/m^2^) Cardiovascular fitness variables: SBP^q^ (mm Hg) and DBP^r^ (mm Hg) A portable spirometer to measure FVC^s^ and FEV1^t^ The Borg RPE^u^ scale was used to determine the exercise intensity	Lower weight and BMI in VGG and AEG in postintervention and follow-up compared to preintervention Improvements in FVC and FEV1 in VGG and AEG in postintervention and follow-up compared to preintervention CG has higher weight and BMI, and worse FVC and FEV1 in postintervention and follow-up vs VGG and AEG
Ji et al (2023) [44]	30 participants with mild to moderate ADHD^v^ (26 males) Age range: 8‐12 years Country: Republic of Korea EG: 16 students (mean age 9, SD 1.46 years) CG: 14 students (mean age 8.85, SD 1.63 years)	Randomized controlled trial 4 weeks EG intervention: 50 min/day, 3 days per week Exergame: Alchemist’s Treasure (D&J Humancare) CG intervention: 50 min/day, 3 days per week using a stationary bike	Anthropometric measures: height (cm), weight (kg), BMI (kg/m^2^) Go/No-go task and EEG^w^ to measure the capacity to sustain attention and response control FAIR^x^ test to assess attention and concentration	Both the EG and CG had significantly increased selective attention, continuous attention, and self-control on the FAIR test Both the EG and CG had significantly reduced response time on the Go/No-go test; for the Go response, the N2 amplitude was increased in Fz in EG but not in CG
Ketelhut et al (2022) [45]	34 students (17 males) Age range: not specified (mean 10.5, SD 0.7 years) Country: Germany EG: 18 students (mean age 10.5, SD 0.7 years) CG: 16 students (mean age 10.5, SD 0.6 years)	2-armed randomized controlled trial 12 weeks EG intervention: 15‐20 min/day, 2 times per week, and normal PE class, 2 times per week Exergame: “Sphery Racer” in Exercube CG intervention: normal PE class, 2 times per week	Anthropometric measures: BMI (kg/m^2^), WHtR^y^ Physical fitness: 20-meter sprint test, using portable electronic timing gates (kettlebell sport); CMJ^z^ using Optojump photocell system, a validated tool for assessing jump height Aerobic fitness: SRT	A significant group-time interaction in CMJ performance, with a significant increase in jumping height in the EG and a significant decrease in the CG Sprint test performance significantly improved in the EG but not in the CG, revealing significant interaction effects and a large effect size Significant group-time interactions and a large effect size were observed for the SRT, with a significant increase in distance covered in the EG
Kolovelonis et al (2023) [46]	122 participants (63 males) Age: not specified (mean 9.98, SD 0.59 years); 4th- to 5th-grade elementary school Country: Greece 1st sample study: 74 participants (36 male) with no exergame experience EG: 38 students CG (waitlist-CG): 36 students 2nd sample study: 48 participants (27 males) with exergame experience	1st study: a 2-group, repeated measures, cross-over quasi-experimental design Single session EG intervention: single exergames PE session Exergame: Just Dance 2015 Console: Xbox One Kinect CG intervention: exergame session after posttest 2nd study: a repeated measures, within-subjects design Single session Single exergame PE session 2 months after a 4-week cognitive intervention with stimulating physical activity games Exergame: Just Dance 2015 Console: Xbox One Kinect	DF^aa^ to assess executive functions according to three conditions: (1) measuring design fluency, (2) measuring inhibition, and (3) measuring cognitive flexibility Situational Interest Scale to assess students’ situational interest according to 4 dimensions (total interest, instant enjoyment, exploration, and novelty)	1st study Exergame effect on EG Significant time-group interaction in the 3 test condition scores (in particular, significant interaction for conditions 1 and 3, no significant interaction for condition 2) and in the total DF score Both groups showed higher scores at time 2 (significant higher improvement for EG) Exergame effect on CG: Significant improvement in total DF score in CG after receiving exergames Significant effect for time (time 2 and time 3 measures) on the 3 conditions’ scores Significant improvement in all 3 test conditions Students’ situational interest: High scores in all dimensions 2nd study: Significant effect in total DF scores from pretest to posttest
Lau et al (2020) [47]	203 participants with intellectual disability (86 males) Age range: 8‐18 years (mean 12.8, SD 2.8 years) Country: Hong Kong EG: 125 students CG: 78 students	Standard 2-arm parallel, single-blinded study 12 weeks EG intervention: two 30-minute sessions per week Exergame: Xbox Sport Season 1 and Sport Season 2 Console: Xbox 360 Kinect CG intervention: usual PA	Anthropometric measures: height (cm), weight (kg), BMI (kg/m^2^), and body fat (%) ActiGraph GT3X accelerometers to assess PA BOT-2 short form to assess motor proficiency	Significant increases in BMI, % body fat, and motor proficiency within both groups at posttest BOT-2^ab^: Significant increase in EG and CG at posttest No significant group differences in BMI, PA levels, or motor proficiency
Liang et al (2020) [48]	87 participants (54 males) Age range: 9‐12 years Country: Hong Kong EG: 30 students (mean age 10.5, SD 0.7 years) CG: 57 students (mean age 10.4, SD 0.8 years)	Quasi-experimental study 8 weeks EG intervention: two 1-hour sessions per week Exergame: Kinect Adventures Console: Xbox 360 Kinect CG intervention: usual PA	Anthropometric measures: height (cm), weight (kg), BMI (kg/m^2^), percentage body fat (%) ActiGraph GT3X-GT3X+ accelerometers to assess PA and sedentary time (min/day) Questionnaires measuring PA-related psychosocial factors (enjoyment, self-efficacy, and social support)	No significant group differences in BMI or body Accelerometers: No significant group differences in daily sedentary time Significant treatment effect on after-school sedentary time Significant group differences on LPA^ac^ Significant treatment effect on LPA and CPM^ad^ No significant group differences between MPA^ae^ and VPA^af^ No significant group differences in PA-related psychosocial factors
McDonough et al (2021) [49]	47 participants (22 males) Age range: not specified (mean 11.8, SD 1.3 years) Country: United States EG: 47 students CG: not used	Cross-sectional study Duration not specified EG intervention: 2 separate 15-minute exergaming sessions first in small groups (n=3 to 4), then in full class (n=23 to 24) Exergame: Xbox One Kinect, Just Dance CG intervention: not used	Anthropometric measures: height (cm), weight (kg), body fat (%) ActiGraph GT3X+accelerometers to measure participants’ duration in SB^ag^, LPA, MVPA^ah^, and steps PA-enjoyment and PA-self-efficacy were evaluated using a 5-point Likert scale	Participants spent more time in SB during the full-class session vs the small-groups session, and more time in MVPA during the small-groups session vs the full-class session Participants had greater enjoyment during the small-groups session vs the full-class session No differences between exergaming sessions for time in LPA and self-efficacy
McGann et al (2020) [50]	40 participants (21 males) Age range: 5‐7 years Country: Republic of Ireland EG: 20 students (mean age 6.3 years) CG: 20 students (mean age 6.4 years)	Randomized trial 8 weeks EG intervention: purpose-built exergames + standard PE (1 hour, once a week, 8 lessons) Four purpose-built exergames developed through Scratch Editor and Kinect 3D sensors: Hop Ball, Jump Ball, Slide Ball, and Alien Attack CG intervention: commercial exergames + standard PE (1 hour, once a week, 8 lessons)	TGMD-2^ai^ to assess locomotor skills	No significant differences between groups at pretest Both groups improved in some locomotor skills at posttest Purpose-built exergames had a significantly stronger effect on all user locomotor outcomes compared to commercial exergames (one locomotor skill)
Medeiros et al (2020) [51]	64 participants (30 males) Age range: 8‐10 years (mean 9.09, SD 0.75 years) Country: Brazil EG: 32 students CG: 32 students	Blind randomized trial 9 weeks EG intervention: two 45-minute sessions per week Exergame: Kinect Sports 1 and 2 and Kinect Adventures Console: Xbox 360 Kinect CG intervention: regular PE classes	TGMD-2 to assess motor skills	Significant improvements in both groups in different motor skills (EG 10/12 motor skills) No significant differences related to the intervention effect between EG vs CG
Mok et al (2020) [52]	3036 participants (1496 males) Age range: 8‐11 years Countries: Croatia, Lithuania, Macedonia, Poland, Romania, Serbia, South Africa, and Turkey EG: 1914 students CG: 1122 students	Quasi-experimental design 10 weeks EG intervention: 3‐5 minute video, twice a day, 5 days per week during class time HOPSports Brain Breaks Physical Activity Solutions’ videos Use of video projectors CG intervention: usual academic activity	Anthropometric measures: BMI (kg/m^2^) APAS to assess PA beliefs, attitudes, and self-efficacy	Significant time-group effect for BMI and all APAS variables except fitness Significant increase in EG attitudes toward PA for self-efficacy and learning when compared to CG
Quintas et al (2020)^aj^ [53]	417 participants (195 males) Age range: 10‐12 years (mean 11.2, SD 1.7 years) Country: Spain EG: 226 students CG: 191 students	Natural experiment with a nonrandomized controlled design 12 sessions for 4 weeks EG intervention: corporal language session + Just Dance Now exergame + productive dance Virtual platform: ClassDojo CG intervention: corporal language session + dancing different dance styles without exergame + productive dance	Perceived Locus of Causality Scale to assess motivation Dispositional Flow Scale 2 to assess flow state Basic Psychological Needs in Exercise Scale (competence, autonomy, and relatedness) Qualitative evaluation of the teacher for Rhythmic Motor Skills (1‐5 points) Qualitative evaluation of the teacher for Commitment and Behavior Learning (1‐5 points)	Better positive gamified exergaming effects on basic psychological needs, commitment to and behavior toward learning and 2 flow dimensions (autotelic experience and time transformation) No interaction effects (time-treatment) in intrinsic motivation, external regulation, and decreased motivation for EG
Quintas-Hijós et al^aj^ (2020) [12]	417 participants (195 males) Age range: 10‐12 years (mean 11.1, SD 1.7 years) Country: Spain EG: 226 students CG: 191 students, 8 teachers (6 males; mean age 37.5, SD 6.12 years)	Natural experiment, nonrandomized controlled design 4 or 6 weeks EG intervention: 135 minutes/week (9 hours total) MDA^ak^ architecture Exergame: Just Dance Now Virtual platform: ClassDojo CG intervention: traditional didactic teaching of dance in Spanish PE	Qualitative measures: field notes and OQQ^al^ Focus groups (n=56 students) Individual semistructured interviews (4 students; 8 teachers) Thematic analysis and COREQ-32^am^ for results analysis	Greatest difficulty expressed: working in groups, complexity of dances, body expression, adapting to the song and the choreography, and making group decisions Frequently shared feelings or opinions: enjoyment, embarrassment, positivity, motivation, fun, creativity, motor learning
Regaïeg et al (2021) [54]	24 participants with intellectual disability (13 males) Age range: 7‐10 years Country: France EG: 12 students (mean age 9.42, SD 1.65 years) CG: 12 students (mean age 8.42, SD 1.08 years)	Quasi-experimental study 10 weeks EG intervention: two 60-minute sessions per week Hybrid program (30-minute virtual game and 30-minute real game) Exergames: Kinect Adventures Consoles: Wii Nintendo and Xbox 360 Kinect CG intervention: adapted football and long jump in PE classes	TGMD-2 to assess FMS^an^	Significant intervention effect and time-group effect on the LS^ao^, OCS^ap^, and GMQS^aq^ No significant group factor effect on the LS, OCS, and GMQS In the posttest, LS is significantly higher in both groups; in addition, OCS and GMQS are significantly higher only in EG compared to CG
Röglin et al (2023) [55]	27 participants (13 males) Age range: not specified (mean 10.5, SD 0.7 years) Country: Germany EG: 27 students CG: not used	Experimental cross-sectional study 12 weeks EG intervention: two 15‐ to 20-minute sessions per week + regular PE classes twice a week (total 135 minutes) Exergame: “Sphery Racer” in the ExerCube CG intervention: not used	Anthropometric measures: height (cm), weight (kg), waist circumference (cm), BMI (kg/m^2^), WHtR, and VO_2_max^ar^ Modified version of the PACES^as^ to measure perceived enjoyment during the ES^at^ and PE class Structured interviews to evaluate staff and teachers’ perceptions of students’ interest, engagement, and motivation during the ES	The mean PACES score during the ES in week 2 was significantly higher than during the PE class in the same week; no significant differences in perceived enjoyment after 2 and 12 weeks of intervention The teachers and study staff reported high levels of interest, motivation, and engagement of most students throughout the ES
San Blas et al (2024) [56]	21 participants (males not specified) Age range: 11‐12 years (mean and SD not specified) Country: Spain EG: 21 students CG: not used	Case study 1 month 6 sessions of 1 hour Each session has 3 parts: anticipated learning (10 minutes), comprehension guide (20 minutes), comprehension consolidation (30 minutes) Exergame: Multiagent Platform	Qualitative data: teachers’ observations about signs of amusement, gestures, or frustration Quantitative data: tool score about the games, Technology Acceptance Model, questionnaire about students’ satisfaction and motivation	Students obtained a slightly lower grade than that obtained from the tool; the tool is able to gain knowledge and reinforcement of the lessons Students agree that the tool is useful in their learning process, and they want to use it in the future Teachers observed that the students attended with greater effort and retained the information more easily, knowing that they would be playing games with the tool
van Biljon et al (2021) [57]	31 participants with overweight or obese (males not specified) Age range: 9‐12 years (mean 11.40, SD 0.86 years) Country: South Africa EG: 11 students VGG: 10 students CG: 10 students	Experimental randomized study 6 weeks EG intervention: 15 minutes, 3 days a week Exergame: Boxing and Hula Hooping Console: Wii Nintendo VGG intervention: 30 minutes, 3 days a week Videogames: Madden NFL 10 and Knockout Kings Console: PlayStation 2 CG intervention: usual activities	Body composition variables: body mass, BMI (kg/cm^2^), and WHR Cardiovascular fitness variables: RHR^au^ (bpm), SBP (mm Hg), DBP (mm Hg), and VO_2_peak^av^ (mL/kg/min)	No significant BMI percentile improvements within the 3 groups Significant WHR decrease in EG, not in VGG and CG Significant RHR improvement in EG, vs VGG and CG Significant VO_2_peak change in the 3 groups No significant SBP and DBP change within the 3 groups
Yamamoto et al (2023) [58]	8 participants (2 males) with FASD^aw^ and ADHD Age range: 9‐13 years (mean 11.4, SD 1.4 years) Country: United States EG: 8 students CG: not used	Uncontrolled trial 6 weeks EG intervention: 50 minutes per session for a total of 12, twice weekly Exergame: Obie exergaming platform (EyeClick Ltd) CG intervention: not used	Anthropometric measures: weight (kg), height (m) 6MWT^ax^ to evaluate walking distance covered in meters after 6 minutes and estimate VO_2_max	6MWT improved from baseline to week 6, which is a 31% improvement in estimated VO_2_max

a Same sample of subjects.

b EG: experimental group.

c CG: control group.

d PE: physical education.

e BRUMS: Brunel Mood Scale.

f APAS: Attitudes Toward Physical Activity Scale.

g PA: physical activity.

h SRT: shuttle run test.

i HDL: high-density lipoprotein cholesterol.

j LDL: low-density lipoprotein cholesterol.

k EHCLG: exergames with high cognitive load group.

l ELCLG: exergames with low cognitive load group.

m DPA: daily physical activity.

n VGG: video game group.

o AEG: aquatic exercise group.

p WHR: waist-to-hip ratio.

q SBP: systolic blood pressure.

r DBP: diastolic blood pressure.

s FVC: forced vital capacity.

t FEV1: forced expiratory volume in the first second.

u RPE: rating of perceived exertion.

v ADHD: attention-deficit/hyperactivity disorder.

w EEG: electroencephalogram.

x FAIR: Frankfurter Aufmerksamkeits-Inventar.

y WHtR: waist-to-height ratio.

z CMJ: countermovement jump test.

aa DF: design fluency.

ab BOT-2: Bruininks-Oseretsky Test of Motor Proficiency—Second Edition.

ac LPA: light physical activity.

ad CPM: counts per minute.

ae MPA: moderate physical activity.

af VPA: vigorous physical activity.

ag SB: sedentary behavior.

ah MVPA: moderate to vigorous physical activity.

ai TGMD-2: Test of Gross Motor Development—Second Edition.

aj Same sample of subjects.

ak MDA: Mechanics-Dynamics-Aesthetics.

al OQQ: open-question questionnaire.

am COREQ-32: Consolidated Criteria for Reporting Qualitative Research.

an FMS: fundamental movement skills.

ao LS: locomotor scores.

ap OCS: object control scores.

aq GMQS: gross motor quotient scores.

ar VO_2_max: maximum oxygen uptake.

as PACES: Physical Activity Enjoyment Scale.

at ES: exergaming session.

au RHR: resting heart rate.

av VO2peak: peak oxygen uptake.

aw FASD: fetal alcohol spectrum disorder.

ax 6MWT: 6-minute walk test.

All studies involve children and adolescents: 23 studies include elementary or middle school children, 1 study includes high school students [39], and another one includes the 8‐ to 18-year-old age group [47]. Most of the articles are fairly balanced between males and females, but sex difference is often not measured in statistical analyses. For 3 studies [40,56,57], the male-female distribution is not known. Most studies include the general population, while 2 studies include children with overweight or obesity [43,57]; 1 study includes children with attention deficit/hyperactivity disorder [44] and another study includes both children with attention deficit/hyperactivity disorder and fetal alcohol spectrum disorder; lastly, 2 studies include children diagnosed with intellectual disability [47,54]. Studies differ in research methodology: there were 6 randomized controlled trials [43-45,50,51,57], 8 nonrandomized controlled studies [12,35,36,38,39,47,53,58], 7 quasi-experimental studies [37,40,41,46,48,52,54], 2 cross-sectional studies [49,55], 1 case study [59], and 1 pilot interventional study [42].

The studies come from different countries, in particular, 2 from North America [49,58], 2 from South Africa [38,57], 4 from South America [35,36,40,51], 6 from Asia [37,39,43,44,47,48], 10 from Europe [12,41,42,45,46,50,53-56], and only one study includes different populations [52].

Thus, their length varied, too, from a minimum of a single session to a maximum of 3 months. In 2 studies, the trial or training duration is not given [40,49]. Regarding the type of exergame used, 7 studies used dance-based exergames, such as Just Dance 2015 on Microsoft Xbox consoles [35,36,40,43,46,49] or Just Dance Now [12,53]. Three studies used videos from the HOPSports Brain Breaks package, which includes different sports such as baseball, basketball, soccer, volleyball, swimming, golf, football, and rowing [37,38,52]. Five studies used exergames based on specific sports such as Nintendo Wii Sports (bowling, boxing, tennis, cycling, table tennis, and basketball) or Wii Fit (yoga, muscle exercises, aerobic exercises, and balance games) or Xbox Sports Season 1 and 2 (including soccer, beach volleyball, bowling, table tennis, track and field, boxing, American football, baseball, darts, golf, skiing, and tennis) [39,43,47,51] or boxing and hula hooping on the Wii Nintendo console [57]. Eight studies used various games involving movement using the Wii balance board (eg, Feline Runner, Hungry Monster, and Whack-a-Slime) [41,54] or Xbox (eg, Kinect Adventures) [48,51] or still other platforms, such as D&J Humancare’s Alchemist’s Treasure [44], Obie [58], or Exercube’s Sphery Racer [45,55]. Three studies developed specific exergames, building on existing ones [42,50,56].

All exergames could be played in a multiplayer mode, for example, by the whole class in some instances or small groups of 3‐4 in others.

The 25 studies were divided into three broad categories according to research objective and outcome measurement:

1. Research studying the effects on psychological or cognitive dimensions. There are 9 papers. The variables under investigation are psychological states or psychosomatic states [35,36]; self-esteem [36]; beliefs and attitudes and self-efficacy with respect to physical activity [37]; attention, concentration and response control, or executive function [44,46]; interest, enjoyment, commitment, flow state, and motivation [46,53,55]. All studies, except for two, found significant effects of exergames on the psychological variables under study. The two studies without significant effects or differences versus the control group specifically addressed intrinsic motivation, external regulation, decreasing amotivation, and perceived enjoyment [53,55].
2. Research studying direct effects on motor and anthropometric aspects or motor skills. There are 12 papers in total. The variables under investigation are physical fitness [38,43,45,57], anthropometric measures [38-40,43,45,47,57,58], physiological factors [39], daily steps [39], and various motor skills [40,41,47,50,51,54,58]. Significant effects of the application of exergames on anthropometric variables were found in 11 papers [38], including papers on children with obesity [43,57], on various motor or locomotor components [40,41,50,51,54,58], on various physiological components [39], and on physical fitness variables [38,45]. In some of these cases [43,51,54], the significant increase in some or all motor skills or anthropometric variables pretest versus posttest did not differ from that of the control group. Only one study on children with intellectual disabilities did not find any effects [47], so exergames were rated as ineffective.
3. Research on the simultaneous effects on motor or physical aspects and psychological variables. There are 4 articles. The variables under investigation include both anthropometric variables [48,49,52], motor skills [48], physical literacy [42], or psychosocial factors such as enjoyment, self-efficacy, and social support [48,49], as well as beliefs and attitudes toward physical activity [52], and improvements in performance and concentration in other school subjects [42]. Significant effects of exergames were found on some anthropometric or motor variables [42,48,49,52], as well as on psychological variables such as attitude toward physical activity, self-efficacy, and learning [52], or motivation, engagement, and concentration in other school subjects [42]. In two studies [48,49], no effects were found on self-efficacy and psychosocial factors.

## Discussion

### Discussion of Included Studies

This scoping review focused on the effect of the use of exergames at school in different countries, during PE lessons and play or sports activities, in order to understand their benefits and advantages and their future applications. For these purposes, 25 articles were included.

Considering the majority of the studies, use of exergames during PE hours and playful sports activities is associated with a number of improvements; more specifically, these devices can provide psychological or cognitive as well as motor benefits for children and adolescents with a median age of 10 years. The studies presented use different types of games and vary in frequency, with an overall average duration of 7 weeks, in line with findings from a recent meta-analysis [31]. Below, the specific effects observed are discussed.

### Discussion on Psychological or Cognitive Effects

We found 4 studies investigating the psychological effects of exergames. Based on them, we reached the conclusion that exergames played during PE hours or during didactic hours can help increase the students’ sense of self-efficacy, vigor, learning, fun, and decrease tension, mental confusion, and perceived anxiety. In addition, they can improve the beliefs and attitudes that children and adolescents have toward sports and physical activities, leading to a general increase in exercise and physical activity [35-37,52]. Also, a review by Joronen and colleagues [59] aimed to observe nonphysical effects of exergames and found that exergames can increase situational interest and enjoyment during physical activity because they are perceived as being more enjoyable. For this reason, children are more likely to carry on playing exergames instead of traditional games. Despite some conflicting results, these researchers reported that successful experiences with exergames could increase perceived self-confidence and consequently the probability of future participation in motor activities [59]. An improvement in self-efficacy and self-confidence was also observed in a previous review and meta-analysis by Andrade and colleagues [60] investigating the effects of exergames in obese children. This could be related to the fact that exergames provide immediate feedback on the student’s actions. The ability to see the outcome of their actions in real time may engage and motivate students to continue the activity.

As far as cognitive abilities are concerned, based on two studies taken into account, it can be concluded that an exergame-based intervention can improve executive-attentive functions, specifically selective attention, self-control, sustained attention, cognitive flexibility, and also the students’ situational interest for the activity [44,46]. Additionally, some exercises with exergames can have positive effects on motivation and concentration in other school subjects, such as mathematics [42].

These improvements can also be observed at a physiological level, with alterations in event-related potential component N2 waves and cortical cingulate area activity, which are visible just after a single intervention [44]. Exergames would appear to better stimulate visual perception thanks to a virtual environment, which may be experienced by students as more dynamic and engaging. This thus suggests that exergames have attractive and interesting features for children, such as the nature of the game played, interactivity, and involvement through sounds and images. These features may increase participation in physical activity.

Although backed by only a few studies, these results were also confirmed in a previous review by Ramírez-Granizo and colleagues [61]: independently of the exergame used, cognitive performance significantly benefited from the sports or physical activity carried out using them. In particular, the use of electronic devices associated with motor activity seems to bring a general improvement in concentration, attention, and reaction speed.

### Discussion on Effects of Motor Abilities

Most of the reviewed studies investigated the influence of exergames on various anthropometric parameters and physical performance. When compared to traditional physical activity interventions, exergames can demonstrably improve motor performance (such as jumping, running, shooting, football, and object control), flexibility and postural stability, and the students’ physical fitness at a general level [40,41,45,51,54]. In addition, exergames can improve muscle mass, decreasing waist circumference and body fat [38,43,47], but they can also lead to a significant reduction in sitting time at weekends [39].

Exergames are effective tools for assisting in the acquisition of motor skills, thanks to the immediate feedback that children receive on their performance. In line with our findings, recent reviews or meta-analyses found that interventions using exergames are effective in developing and improving several components of PE learning or physical fitness, such as agility, balance, and postural stability, while also benefiting the ability to control objects and locomotor skills [31,62]. It must be noted, however, that the authors concluded that their findings were not definitive due to the conflicting outcomes of the studies included in their review.

Despite this variability, most studies support the idea of integrating exergames into traditional PE lessons or using them during teaching hours. This approach can make physical activity more engaging for students while providing variety within the curriculum.

In summary, while current evidence points toward numerous benefits of exergames for improving motor skills and physical fitness among children and adolescents, ongoing research is essential to optimize their application and maximize their potential within educational settings. Further investigations can focus on which games are most effective and establish guidelines for their use to maximize the effectiveness and inclusivity of these tools (such as duration of use, single-player, or group gameplay).

### Strengths and Limitations

As far as we are aware, this is the first scoping review aimed at investigating the motor, psychological, and cognitive benefits of exergame-based interventions in school settings and specifically during PE lessons and play activities.

While the primary strength of our study is its attempt to provide an up-to-date review—considering the last 5 years as a timeframe—of the effects of exergames on motor, psychological, and cognitive abilities, this study is not free of limitations, and, consequently, caution is required when interpreting its results.

First, we only included publications in English, which may have led to the exclusion of further available studies that explored this topic in other languages. Although the studies come from different continents, there is no complete heterogeneity. Second, no systematic review and meta-analysis were conducted to assess the effectiveness of exergames and the generalizability of the results.

Third, the studies considered in the review were highly heterogeneous and had several methodological biases. They used different types of exergames; the length was different as were outcomes and variables. For example, some studies used a single exergaming session [46,49], while other studies included several exergaming sessions, up to 3 months. Also, video games and devices were different across studies, and not all studies had a sufficient sample size or used sample randomization. All this could complicate the interpretation of findings and limit their generalizability.

Thus, more studies, such as systematic reviews and meta-analyses, are encouraged to reinforce the validity of the present findings.

### Conclusions

In our scoping review of the effects of exergames on motor, psychological, and cognitive abilities during PE lessons and playful sports activities, we focused on the school context, given the central and important role that schools currently have in promoting physical activity in children and adolescents [11].

Our findings show that exergames can have a beneficial effect on children’s motor skills, cognitive flexibility and attentional functions, and overall well-being, improving the sense of self-efficacy, self-confidence, and mood during PE lessons.

This may have significant implications for public health or education: exergames may become accessible and useful devices for promoting physical activity in young people, potentially bringing benefits not only on a motor skill level but also on a psychological and cognitive level, increasing children’s participation in physical activities and leading to a general improvement in their sense of self-efficacy and well-being.

However, lack of knowledge of their potentials and benefits hinders their wider application. Furthermore, despite their effectiveness, their use is not yet common in the educational and school context.

For this reason, a more systematic application of digital technology in the school setting would contribute to improving and designing new immersive, effective, and inclusive interventions aimed at increasing the physical activity and well-being of children and adolescents. Exergames can improve children’s physical and cognitive skills, enjoyment, and involvement in exercise and general well-being, thus becoming a complementary and additional device to traditional PE exercises and helpful tools to increase motor activity during extracurricular activities.

## Supplementary material

10.2196/71416 Multimedia Appendix 1 Search strategies.

10.2196/71416 Checklist 1 PRISMA-ScR checklist.

## Abbreviations

PCC: population, concept, and context
PE: physical education
PRISMA-ScR: Preferred Reporting Items for Systematic Reviews and Meta-Analyses Extension for Scoping Reviews
WHO: World Health Organization
