# Supplementary material for: Effects of Exergames on Motor Skills, Psychological Well-Being, and Cognitive Abilities in Schoolchildren and Adolescents: Scoping Review
Source: JMIR Pediatr Parent. 2025 Sep 17;8:e71416. doi: 10.2196/71416 (PMC12443355; doi:10.2196/71416)
Supplement: Multimedia Appendix 1 [file pediatrics-v8-e71416-s001.docx]

### **Search Results for Each Source**

PUBMED

("Physical Exercise" OR "Physical Education" ) AND ("Exergaming" OR "Active Video Games" OR "exergames" OR "interactive videogame" OR "technology") AND ("Children" OR "Adolescent") AND ("school" OR "school education" OR "education") NOT (Meta-Analysis OR Review)

WEB OF SCIENCE

("Physical Exercise" OR "Physical Education" ) AND ("Exergaming" OR "Active Video Games" OR "exergames" OR "interactive videogame" OR "technology") AND ("Children" OR "Adolescent") AND ("school" OR "school education" OR "education") NOT (Meta-Analysis OR Review)

SCOPUS

(TITLE-ABS-KEY ( physical AND exercise ) OR TITLE-ABS-KEY ( physical AND education ) AND TITLE-ABS-KEY ( exergaming ) OR TITLE-ABS-KEY ( active AND video AND games ) OR TITLE-ABS-KEY ( exergames ) OR TITLE-ABS-KEY ( interactive AND videogame ) OR TITLE-ABS-KEY ( technology ) AND TITLE-ABS-KEY ( children ) OR TITLE-ABS-KEY ( adolescent ) AND TITLE-ABS-KEY ( school ) OR TITLE-ABS-KEY ( school AND education ) OR TITLE-ABS-KEY ( education ) AND NOT TITLE-ABS-KEY ( meta-analysis ) OR TITLE-ABS-KEY ( review ) ) AND PUBYEAR > 2018 AND PUBYEAR < 2025 AND ( LIMIT-TO ( DOCTYPE , "ar" ) )
